# Autosomal Dominant Alport Syndrome Diagnosed in an Elderly Man

**DOI:** 10.7759/cureus.94725

**Published:** 2025-10-16

**Authors:** Reza Khorsan, Farid Arman, Mrinalini Sarkar

**Affiliations:** 1 Medicine/Nephrology, University of California Los Angeles, Los Angeles, USA

**Keywords:** alport syndrome, autosomal dominant, chronic kidney disease, genetic disorders, genetic kidney disease

## Abstract

Autosomal dominant Alport syndrome (ADAS) is an uncommon diagnosis and is often missed in patients with chronic kidney disease of unknown origin unless specific genetic testing is performed. It results from heterozygous mutations in the COL4A3 and COL4A4 genes. The clinical presentation is variable but generally milder than that of X-linked or autosomal recessive Alport syndrome. In this article, we present the case of a 78-year-old man who was diagnosed with ADAS during evaluation in a nephrology clinic. We also discuss the condition and the challenges related to the terminology surrounding Alport syndrome.

## Introduction

Autosomal dominant Alport syndrome (ADAS) is a condition that many nephrologists are not familiar with. Most practitioners recognize the classical X-linked form of Alport syndrome, which in males causes progressive chronic kidney disease, bilateral sensorineural hearing loss, and ocular abnormalities. First described in 1927 [1], Alport syndrome accounts for approximately 1%-2% of end-stage renal disease (ESRD) cases in Europe [2]. It is caused by mutations in the COL4A5 gene [3], leading to type IV collagen defects and abnormalities of the glomerular, cochlear, and ocular basement membranes. The glomerular basement membrane consists of a network of type IV collagen that serves as a selective filtration barrier; mutations in its alpha chains disrupt this network, resulting in basement membrane defects [2,3]. The autosomal recessive form of Alport syndrome, caused by homozygous mutations in the COL4A3 or COL4A4 genes, accounts for fewer than 15% of cases [4,5]. This form produces a phenotype similar to the X-linked variant but affects males and females with equal severity. ADAS, on the other hand, results from heterozygous mutations in COL4A3 or COL4A4 and typically causes a milder phenotype [6-10]. Some individuals with known mutations remain asymptomatic or present only with microscopic hematuria, while others progress to require kidney replacement therapy (KRT) [7-9]. Mild cases have also been described as “familial benign hematuria” or “thin basement membrane disease” [11,12]. Many elderly patients with chronic kidney disease may unknowingly have this condition unless specific genetic testing is performed.

## Case presentation

A 78-year-old man with stage 3a chronic kidney disease and hypertension was referred to the nephrology clinic for further evaluation and management. His medications included rosuvastatin for hyperlipidemia and metoprolol succinate for hypertension. He had no history of ocular abnormalities but had been diagnosed with sensorineural hearing loss 10 years earlier, at age 68. The patient also had bilateral renal cysts and a long-standing history of microscopic hematuria. He denied any episodes of gross hematuria or kidney stones. A previous cystoscopy was normal, and a CT urogram showed no pathological lesions. He had never undergone a kidney biopsy. The patient’s brother had a diagnosis of polycystic kidney disease (PCKD) and underwent renal transplantation in his 70s; he was later deceased.

**Figure 1:**
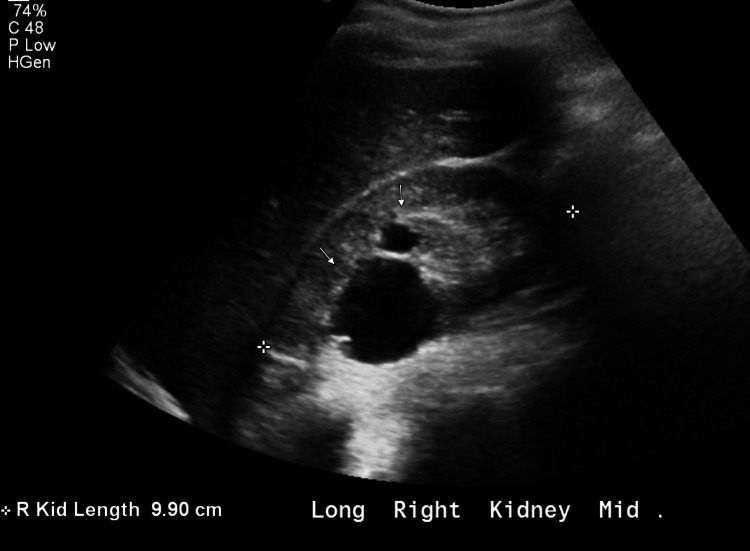
Longitudinal view of the right kidney showing mild cortical thinning and evidence of cysts (white arrows)

**Figure 2:**
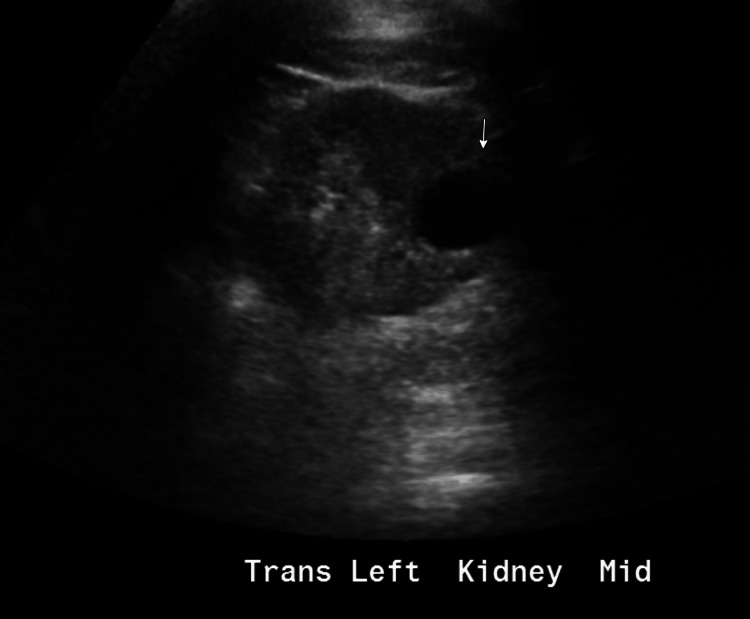
Transverse view of the left kidney demonstrating a subcortical cyst (white arrow)

At the time of evaluation, the patient’s serum creatinine was moderately elevated. Urine dipstick analysis revealed microscopic hematuria without proteinuria (Table 1). Kidney ultrasound showed a right kidney measuring 9.4 cm with mild cortical thinning and a left kidney measuring 8.1 cm with mildly increased echogenicity. Several bilateral renal cysts were also noted (Figure 1 and Figure 2).

**Table 1:** Initial laboratory values

Laboratory tests	Value	Normal value
Serum creatinine	1.8 mg/dL	0.6-1.3 mg/dL
Urine analysis dipstick protein	Negative	Negative
Urine analysis dipstick blood	Trace	Negative

Seven years prior, his serum creatinine was 1.5 mg/dL but had been checked infrequently since. There was concern for progressive chronic kidney disease, possibly secondary to PCKD. A Natera Renasight genetic panel (saliva test) was obtained for further evaluation. The results were positive for a heterozygous mutation in the COL4A4 gene, consistent with ADAS. The variant consisted of a single amino acid substitution (missense) of glycine (Gly) to glutamine (Glu) at codon 408 in exon 20 (p.Gly408Glu), located within a specific triple-helix repeat domain, disrupting the Gly-X-Y motif. According to the Renasight report, this rare variant has been observed in less than 0.01% of cases in the Broad gnomAD dataset. The patient was referred to a local Alport syndrome clinic for genetic counseling, educational resources, and discussion of treatment options.

## Discussion

ADAS is a diagnosis that is often overlooked as a cause of chronic kidney disease. Once thought to be extremely rare, it is now believed to be more common than previously recognized [13]. The estimated prevalence largely depends on how the condition is defined. Historically, it was diagnosed only when significant chronic kidney disease developed; however, more recently, ADAS has been accepted as present when a pathogenic mutation is identified in a COL4A3 or COL4A4 gene. In such cases, carriers may exhibit only minimal urinary abnormalities, such as microscopic hematuria, while others may develop more advanced kidney disease characterized by proteinuria and progressive decline in eGFR [14]. Differentiating ADAS from other causes of chronic kidney disease, such as polycystic kidney disease (PCKD) or hypertensive nephrosclerosis, can be challenging without genetic testing.

In one of the largest studies of ADAS, a Spanish cohort involving 82 families and 252 patients with pathogenic COL4A3 or COL4A4 mutations, microscopic hematuria was the most common finding [15]. Proteinuria was the second most frequent feature. The median kidney survival in this cohort was 67 years. As reported in other studies, there was significant interfamilial and intrafamilial variability in disease expression [14-16]. Nephrotic-range proteinuria occurred in only three patients. Among those who underwent kidney biopsy, findings included thickening, thinning, and splitting of the glomerular basement membrane, while a minority demonstrated focal segmental glomerulosclerosis (FSGS). Kidney survival did not correlate with any specific gene variant. In this and other cohorts, sensorineural hearing loss and ocular abnormalities were infrequent, features more commonly associated with X-linked or autosomal recessive Alport syndrome (ARAS). These findings underscore the broad clinical spectrum of ADAS, even among families with known advanced CKD or individuals requiring KRT. As illustrated in the present case, the study emphasizes the importance of genetic testing in patients with CKD of uncertain etiology.

Table 2 summarizes the different forms of Alport syndrome.

**Table 2:** Comparison of clinical properties of different types of Alport syndrome ARAS: autosomal recessive Alport syndrome, ADAS: autosomal dominant Alport syndrome, COL4A3: collagen 4 alpha 3. COL4A4: collagen 4 alpha 4, COL4A5: collagen 4 alpha 5, ESRD: end-stage renal disease.

Alport syndrome type	Genes	Microhematuria	Proteinuria	Average decade reaching ESRD	Sensorineural hearing loss	Ocular abnormalities
X-linked	COL4A5	+++	+++	Third (male)	+++	+++
ARAS	COL4A3, COL4A4	+++	+++	Second	+++	+++
ADAS	COL4A3, COL4A4	+++	+	Seventh	+	+

Currently, no specific therapy exists for ADAS. The mainstay of management, as in other forms of CKD, is renin-angiotensin-aldosterone system (RAAS) inhibition, which has demonstrated benefit in other types of Alport syndrome [17,18]. Although specific evidence is lacking, sodium-glucose cotransporter 2 (SGLT2) inhibitors may also prove beneficial, along with optimal management of hypertension and obesity. Genetic counseling is strongly recommended to facilitate screening of family members and enable early intervention before the onset of advanced kidney disease. 

## Conclusions

In patients presenting with chronic kidney disease of unknown cause and microscopic hematuria, nephrologists should consider genetic testing for ADAS. Most of these patients will not exhibit the ocular or hearing abnormalities classically associated with X-linked Alport syndrome. In this case, the patient had sensorineural hearing loss diagnosed at age 68, which was more likely age-related. It is also plausible that his brother had the same condition as the underlying cause of his ESRD and was misdiagnosed as having PCKD, although this remains unconfirmed, as no genetic testing was performed. Identifying such patients may allow for the screening of relatives and early intervention. To further delineate the relationship between COL4 gene mutations and CKD phenotypes, broader family genotyping would be beneficial. In select cases, kidney biopsies of index patients and relatives could be correlated with genetic findings, though such studies may be logistically challenging.

The nephrology community should also aim to clarify and standardize the terminology surrounding these genetic disorders, as Alport disease or Alport syndrome often implies a condition with ocular and cochlear abnormalities in addition to renal disease, which may not reflect the full clinical spectrum. Adopting terminology such as "collagen type 4 disease" with specification of inheritance pattern, and moving away from older terms like "thin basement membrane "disease and "benign familial hematuria," may help reduce confusion and improve diagnostic accuracy.
